# Differing long term trends for two common amphibian species (*Bufo bufo* and *Rana temporaria*) in alpine landscapes of Salzburg, Austria

**DOI:** 10.1371/journal.pone.0187148

**Published:** 2017-11-09

**Authors:** Martin Kyek, Peter H. Kaufmann, Robert Lindner

**Affiliations:** Haus der Natur, Museum für Natur und Technik, Museumsplatz 5, Salzburg, Austria; Universitat Trier, GERMANY

## Abstract

This study focuses on the population trends of two widespread European anuran species: the common toad (*Bufo bufo*) and the common frog (*Rana temporaria*). The basis of this study is data gathered over two decades of amphibian fencing alongside roads in the Austrian state of Salzburg. Different statistical approaches were used to analyse the data. Overall average increase or decrease of each species was estimated by calculating a simple average locality index. In addition the statistical software TRIM was used to verify these trends as well as to categorize the data based on the geographic location of each migration site. The results show differing overall trends for the two species: the common toad being stable and the common frog showing a substantial decline over the last two decades. Further analyses based on geographic categorization reveal the strongest decrease in the alpine range of the species. Drainage and agricultural intensification are still ongoing problems within alpine areas, not only in Salzburg. Particularly in respect to micro-climate and the availability of spawning places these changes appear to have a greater impact on the habitats of the common frog than the common toad. Therefore we consider habitat destruction to be the main potential reason behind this dramatic decline. We also conclude that the substantial loss of biomass of a widespread species such as the common frog must have a severe, and often overlooked, ecological impact.

## 1. Introduction

Amphibian species are declining worldwide and in Europe, in fact they are considered the most endangered class of vertebrates [1]. The reasons for this are various and often cumulative. Among them are habitat degradation, habitat loss, road mortality, introduction of predatory fish, diseases as well as climate change [1, 2, 3, 4].

Even though there are many studies dealing with amphibian conservation, the growing concern about declining amphibian populations was often based on either anecdotal evidence or derived from limited, often short-term data sets [5, 6]. In the last years though, increasing efforts were undertaken to generate new, or analyse existing, long-term data sets, often derived from voluntary conservation work [7, 8, 9, 10]. These studies increased our knowledge about natural population fluctuations in amphibians [7] and showed the dimension of local declines for different species [9, 10, 11]. They also raised questions about species or habitat specific differences in population dynamics [7, 10].

The focus of conservation concerns and efforts (such as monitoring and population-level ecological studies) often lies on rare or endemic species threatened by immediate extinction rather than on common and widespread species. This leads to a conservation bias that tends to overlook species that are important for ecosystem services especially because of their contribution to overall biomass. Even a small population decline of such species leads to a substantial loss in biomass and can lead to recognizable changes in ecosystems, true to the motto: “common species shape the world” [12, 13].

As in the recent analyses of Petrovan and Schmidt [10], our study focuses on two syntopic European anuran species, which are both still widely distributed throughout most parts of Central-Europe and the Alps: the common toad (*Bufo bufo*), and the common frog (*Rana temporaria*). These two species are the two most common amphibian species all over Central Europe [14, 15]. They are both considered species of “least concern” in the recent IUCN Red List [16]. This suggests that the populations of these common species are stable. A closer look, however, sometimes reveals dramatic changes in local populations. This is reflected in an increment in conservation concern from worldwide to national and regional levels. In Austria both species are considered as “near threatened” at a national scale [17], in the state of Salzburg the common toad is even considered as “vulnerable” [18].

The dense existing, and still growing, road network in Central-Europe is a serious threat for all amphibian species and leads to increased isolation of populations [1, 19, 20]. Both studied species are so called “explosive breeders” with a short reproductive season in early spring. They undertake migrations between their terrestrial habitat and spawning sites. In the worst case, roads separate terrestrial habitats from breeding ponds and can pose a serious threat to local amphibian populations. To reduce road mortality amphibian fences, as well as permanent tunnel systems, have been, and are being, installed in many places throughout Europe. The basis of this study is data gathered over two decades of amphibian fencing alongside roads in the Austrian state of Salzburg, highlighting that these fences are not just an important instrument for applied nature conservation, but also generate valuable long term data on population trends (compare also [10]). These fences have been maintained all over Salzburg covering different landscapes with varying intensities of agricultural land use. Our study focuses on three synthesising aims: (1) to test whether this long-term data set gathered through volunteer amphibian fencing allow us to derive any significant population trends, (2) to compare the different statistical analysis methods used and (3) to discuss the potential ecological reasons for any ascertained trends. The available data set makes it possible not only to compare two species, but also to scrutinize differences at the landscape level.

## 2. Study area

**Fig 1:**
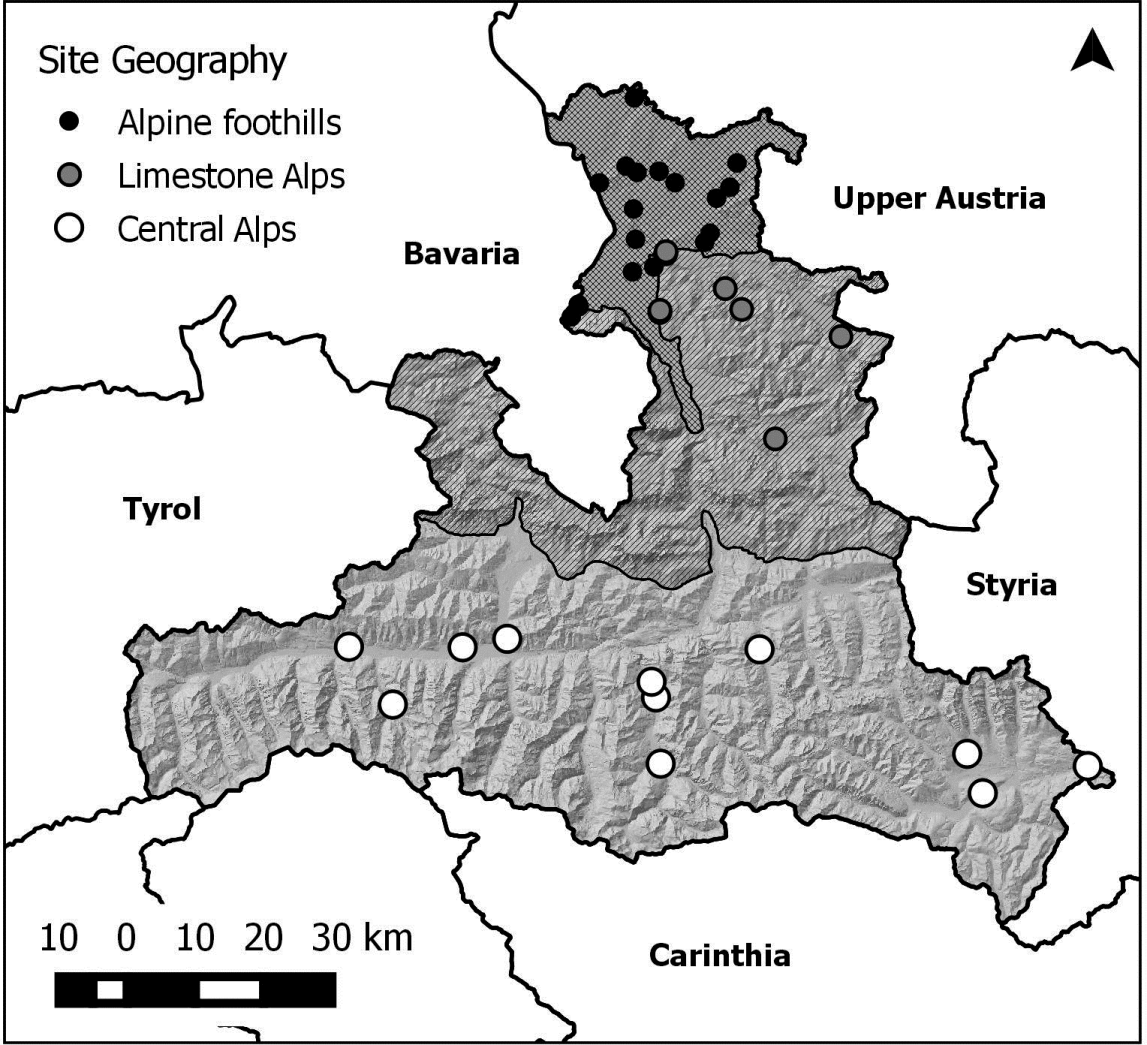
Map of the Austrian state of Salzburg, location of the monitored sites and their categorization based on major geographical regions. Created using maps from Land Salzburg—data.salzburg.gv.at under the CC BY 3.0 AT license, original copyright 2016.

The state of Salzburg (Austria) is located on the northern border of the Alps and stretches over an area of 7,156 km^2^. Due to its geography and geology Salzburg can be divided into three major regions: the Alpine foothills in the north (approx. 300–700 m above sea level), the limestone Alps (approx. 500–2,600 m) and the Central Alps (approx. 550–3,600 m) in the south (see Table 1 and Fig 1).

**Table 1:** List of sites.

#	name	X	Y	elevation	geography	time	⌀ B.bufo	slope (B.b.)	⌀ R.temp.	slope (R.t.)
1	Weitwörth	348414	5308082	410	AF	1995–2015	157	0.043	22	**-0.024**
2	Sinnhubstrasse	353263	5295053	430	AF	2005–2015	1468	**-0.067**	55	**-0.086**
3	Michaelbeuern	353537	5320544	440	AF	1995–2005	202	**-0.199**	30	**-0.111**
4	Kohlgraben	345323	5290286	490	AF	1995–2015	113	**-0.006**	305	0.003
5	Glanegger Straße	345446	5289955	500	AF	2005–2015	1829	**-0.003**	1981	0.068
6	Hinterreith	344283	5288458	520	AF	2001–2015	244	0.102	362	0.065
7	Kreuzbergpromenade	356388	5295763	540	AF	2012–2015	231	0.314	62	0.689
8	Wirthenstätten	359501	5308122	550	AF	2010–2015	1239	0.070	321	0.159
9	Luginger See	353420	5304264	550	AF	2014–2015	372	**-0.148**	1963	0.073
10	Einkehrstall	357161	5309771	560	AF	2002–2012	291	**-0.166**	10	0.096
11	Bergheim Plainstraße	353705	5299818	560	AF	2006–2015	107	0.077	32	0.280
12	Henndorf Altentann	365584	5305834	560	AF	2012–2015	102	0.023	984	**-0.212**
13	Neumarkt Sighartstein	368573	5310995	580	AF	2012–2015	30	**-0.407**	270	0.029
14	Plainfeld Seitenfeld	364625	5300720	590	AF	2011–2015	329	**-0.070**	-	0.000
15	Plainfeld Hub	363843	5299387	600	AF	2011–2015	4	0.167	41	0.762
16	Kienberg	367541	5307446	610	AF	2014–2015	41	**-1.024**	408	0.199
17	Obertrum, Hohengarten	353922	5309625	620	AF	2002–2015	263	0.046	16	0.093
18	Obertrum, Au	352318	5310531	650	AF	2012–2015	207	**-0.156**	21	**-0.420**
19	Sankt Jakob	357290	5289403	500	LA	2002–2015	637	**-0.023**	631	**-0.049**
20	Sankt Jakob, G. Buchmayr	357279	5289090	510	LA	2009–2015	281	**-0.098**	281	**-0.098**
21	Guggenthal B158	358195	5298012	610	LA	1998–2013	133	0.149	789	**-0.024**
22	Guggenthal Gaisberg	358122	5297696	640	LA	1996–1998	15	0.000	468	**-0.069**
23	Strobl	383814	5285560	660	LA	1998–2015	488	**-0.007**	15	0.019
24	Abtenau	374195	5270567	670	LA	1995–2015	651	**-0.008**	394	**-0.036**
25	Faistenau, Bramsau	366852	5292594	670	LA	2014–2015	129	**-0.837**	60	**-0.233**
26	Hintersee	369267	5289550	690	LA	1995–2015	2978	**-0.050**	1218	**-0.192**
27	Thumersbacher Straße	334909	5241267	750	CA	1995–2005	8581	0.094	1545	**-0.017**
28	Mittersill Burgwies	311707	5240104	780	CA	2001–2015	277	**-0.138**	34	**-0.258**
29	Piesendorf	328370	5240023	790	CA	2001–2015	553	0.044	563	**-0.233**
30	Gastein Maierhofen	356062	5234978	820	CA	1995–2006	7	0.000	483	**-0.045**
31	Gastein Patschgwiese	356703	5232668	830	CA	1995–2005	110	0.000	8782	**-0.008**
32	Gastein Bertahof	357393	5222966	850	CA	1995–2008	76	0.169	648	**-0.100**
33	Kleinarl	371886	5239729	940	CA	1995–2008	10	**-0.737**	1319	**-0.084**
34	Stubachtal	318142	5231635	980	CA	2004–2015	1667	**-0.092**	22	**-0.055**
35	Weißpriach	402311	5224411	1000	CA	1995–2005	39	0.325	5880	**-0.016**
36	Unternberg	404605	5218645	1020	CA	2005–2015	1227	0.180	45	**-0.070**
37	Seethalersee	419986	5222612	1240	CA	2002–2015	902	0.058	741	**-0.090**

Sitename, coordinates (WGS84 UTM 33N), elevation, geographical categorization (AF = Alpine foothills, LA = Limestone Alps, CA = Central Alps), time frame of monitoring, mean number of individuals per year and slope of linear regressions for both species common toad (*B*.*bufo*) and common frog (*R*.*temp*.). Bold numbers indicate a negative trend.

The state of Salzburg is highly developed with intensive agricultural exploitation even in the Alpine areas. A dense road network, with a length of about 9,000 km, spans over the lowlands and into the mountain valleys (own calculation based on data from local government sources and Openstreetmap).

Although there are no quantitative data, many years of field experience of the authors indicate that the habitat features for amphibians of the three regions differ substantially. The alpine foothills are characterized by a moderate habitat quality with intensive agriculture and only a few remaining extensive wetland areas. In comparison, the habitat quality of the limestone Alps is much better, with more extensive land use in hilly and montane areas. In the central Alps most of the amphibian populations are located in the valley bottoms which are again characterized by relatively intensive land use. This is a consequence of the alpine topography which leads to a concentration of human activities in the scarce utilizable lowland areas. Here in the central Alps the amphibian populations of the valleys are also isolated by mountain ranges reaching well above 2,800 m. This effect is further intensified by the dense road network. The limestone Alps on the other hand are less affected by these geographical and anthropogenic influences. The main reasons for population isolation in the alpine foothills are due to intensive agriculture and traffic.

A total of 37 amphibian migration routes crossing roads in Salzburg have been protected by fencings and monitored over the last 21 years: 18 of these are within the Alpine foothills, 8 in the Northern Limestone Alps and 11 in the Central Alps (Fig 1).

## 3. Methods

### Data collection

The data were collected from 1995 to 2015 using temporary amphibian fencing, with pitfall traps, alongside roads. Fences were installed every year after the initial thawing period to intercept the spring breeding migration of amphibians. Fence material and installation was implemented according to official guidelines [21, 22, 23]. Fences were at least 40 cm in height and buried into the ground. Pitfall traps were installed with a maximum interval of 30 meters to each other.

Daily maintenance of the pitfall traps and amphibian census was carried out in the early morning by volunteers who were instructed in species identification each year. Fences were dismantled after spring migration if no amphibians were registered in three consecutive nights despite appropriate weather conditions, usually after 4–6 weeks.

State wide data collection has been carried out since 1995. In the year 2001 a government financed coordinator was assigned to supervise fence installation, assist the volunteers and collect data on numbers of migrating amphibians. The numbers of collected amphibians were recorded in a spreadsheets, for each year, species and location. The data were collected at the biodiversity database of the museum “Haus der Natur”, Salzburg.

The number of amphibian fences in Salzburg changed over time. New migration sites were discovered and incorporated in the protection and monitoring system. Some sites were abandoned because of low abundances. At some sites permanent mitigation measures in the form of tunnel systems, with permanent guide walls in between, were installed. Monitoring at such sites did not continue.

### Data preparation

Only counts based on comparable methods were accounted at each site. Sites and years in which no monitoring took place (due to missing maintenance or installation of permanent tunnel-systems) were treated as missing data rather than zero.

For this study only the numbers of those individuals which were migrating towards their breeding ponds were taken into account. Individuals moving back to their terrestrial habitat were not counted. In two cases (two years at one site) where the numbers of arriving and departing individuals were not counted separately, these data were not taken into account and treated as missing.

On one site the partial installation of a tunnel system, in combination with a fence, made the monitoring conditions not comparable to the previous years. The data set after installation was also deleted and treated as missing data, prior to analysis.

On various sites other amphibian species were also registered (e.g. *Ichthyosaura alpestris*, *Lissotriton vulgaris*, *Rana dalmatina*) but in much smaller numbers, not allowing the derivation of any statistical significant state-wide trends. These species were, therefore, not considered in this study.

The simple sum of counted individuals in absolute numbers can not represent actual population trends. The main reason therefore is the different number of monitored sites over the sample period. The number of individuals is dependent on the number of monitored sites. A population trend is therefore not directly apparent based on the raw numbers.

### Average locality index (ALI)

The calculation of the average locality index (ALI) as described by Loman & Andersson [7] allows us to illustrate the average population change over the years, even though the numbers of monitored migration sites and, therefore, the total number of counted individuals differ from year to year.

First we calculate a locality index (LI_ys_) by dividing the number of individuals counted for each species, site and year (N_ys_) by the average number counted for this species at this site (⌀N_s_):

$$LI_{ys}=N_{ys}/⌀N_{s}$$

This index produces a number below 1.00 in years with a relatively low count of individuals and a number above 1.00 in years with a relatively high count of individuals. This allowed us to compare yearly statistics between big and small populations.

Then we calculated the average locality index (ALI_y_) across all sites for each year, as to be able to present the overall population trend for each species:

$$ALI_{y}=⌀\;LI_{ys}$$

This index is independent of the number of sites monitored each year. Finally the linear trend of the ALI was calculated to summarise the trend of counted numbers for each species. This simple and conservative method visualises an average trend in counted numbers for each species, yet offers no test-statistics like standard errors or significances.

### TRIM

As a second approach to analyse the population trends we used the statistical software TRIM (TRends & Indices for Monitoring data, Version 3.53; [24]). This software makes better use of the available data by imputing missing counts, calculating standard errors and offering various test-statistics. Missing values are imputed using log-linear Poisson regressions. TRIM is, furthermore, capable of categorizing data by covariates and testing their effects on the observed changes, using Wald tests [25].

Due to highly different population sizes our counts are not Poisson distributed. Goodness of fit tests could therefore not be used [24]. Hence the different test runs were compared on the basis of Wald tests and standard deviations.

Model selection included linear and time-effect-models as well as different categorization based on the location of the migration sites: no covariates, three geographical covariates (Alpine foothills, Northern Limestone Alps and Austrian Central Alps, see Fig 1). In another model we pooled the Limestone and Central Alps into one “alpine” covariate (model selection and test statistics see Tables 2 and 3).

**Table 2:** Test statistics for the different TRIM-models for the common toad: AIC, Wald-test, mean standard errors of the overall imputed indices and overall slope of the model. The classification of the trend follows Pannekoek & van Strien (2005): stable = no significant increase or decline, and it is certain that trends are less than 5% per year. Covariate groups based on geographic location: 2 covariates (Alpine foothills, alpine), 3 covariates (Alpine foothills, Limestone Alps, Central Alps).

Model	AIC value	Wald-Test Covariates	mean std.err.	Overall slope model
Time Effects	77808.89	-	0.290	Stable
Linear Trend	78465.22	-	0.218	Stable
Time Effects 2 Covariates	71998.86	22.14, df = 20, p = 0.3327	0.423	Stable
Linear Trend 2 Covariates	74550.79	20.03, df = 5, p = 0.0012	0.225	Stable
Time Effects 3 Covariates	54954.93	95.29, df = 40, p = 0.0000	0.395	Stable
Linear Trend 3 Covariates	58961.68	95.81, df = 12, p = 0.0000	0.213	Stable

**Table 3:** Test statistics for the different TRIM-models for the common frog: AIC, Wald-test, mean standard errors of the overall imputed indices and overall slope of the model. The classification of the trend follows Pannekoek & van Strien (2005): moderate decline = significant decline, but not significantly more than 5% per year. Covariate groups based on geographic location: 2 covariates (alpine foothills, alpine), 3 covariates (Alpine foothills, Limestone Alps, Central Alps).

Model	AIC value	Wald-test covariates	mean std.err.	Overall slope model
Time Effects	92481.79	-	0.136	Moderate decline (p<0.01)
Linear Trend	93465.97	-	0.086	Moderate decline (p<0.01)
Time Effects 2 Covariates	76374.10	41.08, df = 20, p = 0.0036	0.171	Moderate decline (p<0.01)
Linear Trend 2 Covariates	78835.74	46.81, df = 5, p = 0.0000	0.104	Moderate decline (p<0.01)
Time Effects 3 Covariates	65437.47	90.16, df = 40, p = 0.0000	0.135	Moderate decline (p<0.01)
Linear Trend 3 Covariates	68168.88	100.98, df = 14, p = 0.0000	0.098	Moderate decline (p<0.01)

To rule out a potential bias within the dataset we also calculated TRIM models excluding those eight migration sites on which data collection stopped due to installation of permanent tunnel systems.

## 4. Results

### Overview

Thirty-seven different amphibian migration sites were monitored between 1995 and 2015. The sites were monitored from 2 up to 21 years (average 10 years). Eight of these migration sites have been protected partially or entirely by a permanent tunnel-system in the last decade, and were therefore not monitored afterwards. On two sites maintenance was discontinued due to low numbers. Altogether there were 324,681 common toads and 302,067 common frogs recorded in pitfalls along the fences over those 21 years.

**Fig 2:**
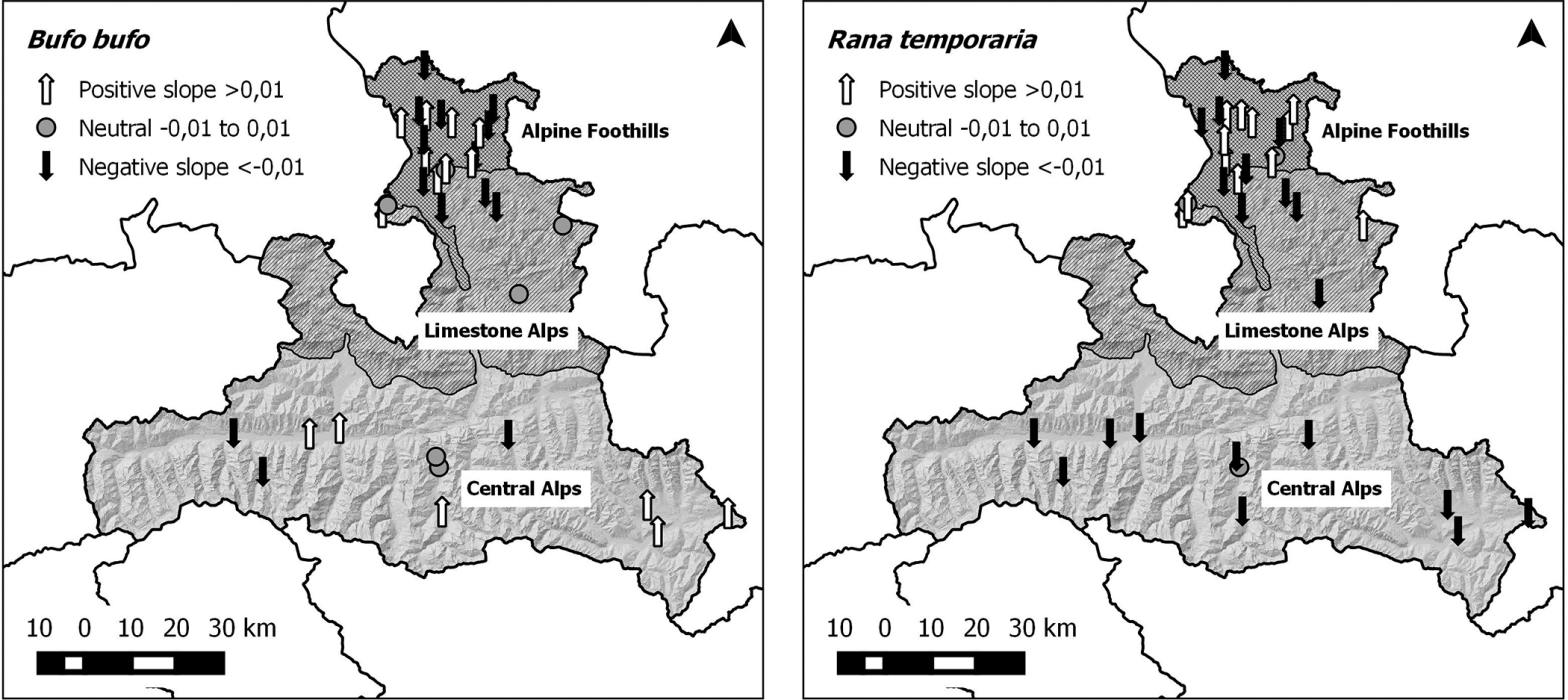
Categorization of study-sites based on increasing (positive slope), stable (neutral) and decreasing (negative slopes) counts based on linear regressions of the absolute counts for each species. Created using maps from Land Salzburg—data.salzburg.gv.at under the CC BY 3.0 AT license, original copyright 2016.

The mean number of migrating individuals per year and site spans from 4 up to 8,581 common toads and from 10 up to 8,782 common frogs. At one site only common toads and no common frogs were registered. The statistical slope of each site and species allows a rough categorization in declining (<0) and increasing (>0) populations (see Table 1 and Fig 2).

In Fig 2 we present the regression slope for each site and species as well as their location within the state of Salzburg. The common toad shows a heterogeneous pattern, with both positive (49%) and negative trends (51%) distributed over the three different geographical regions. On the contrary, the common frog shows no increasing trend over the whole Central Alps, an overall higher number of decreasing sites (62%) and bigger decreases at sites with higher abundances (compare Table 1).

### Analysis based on average locality indices

**Fig 3:**
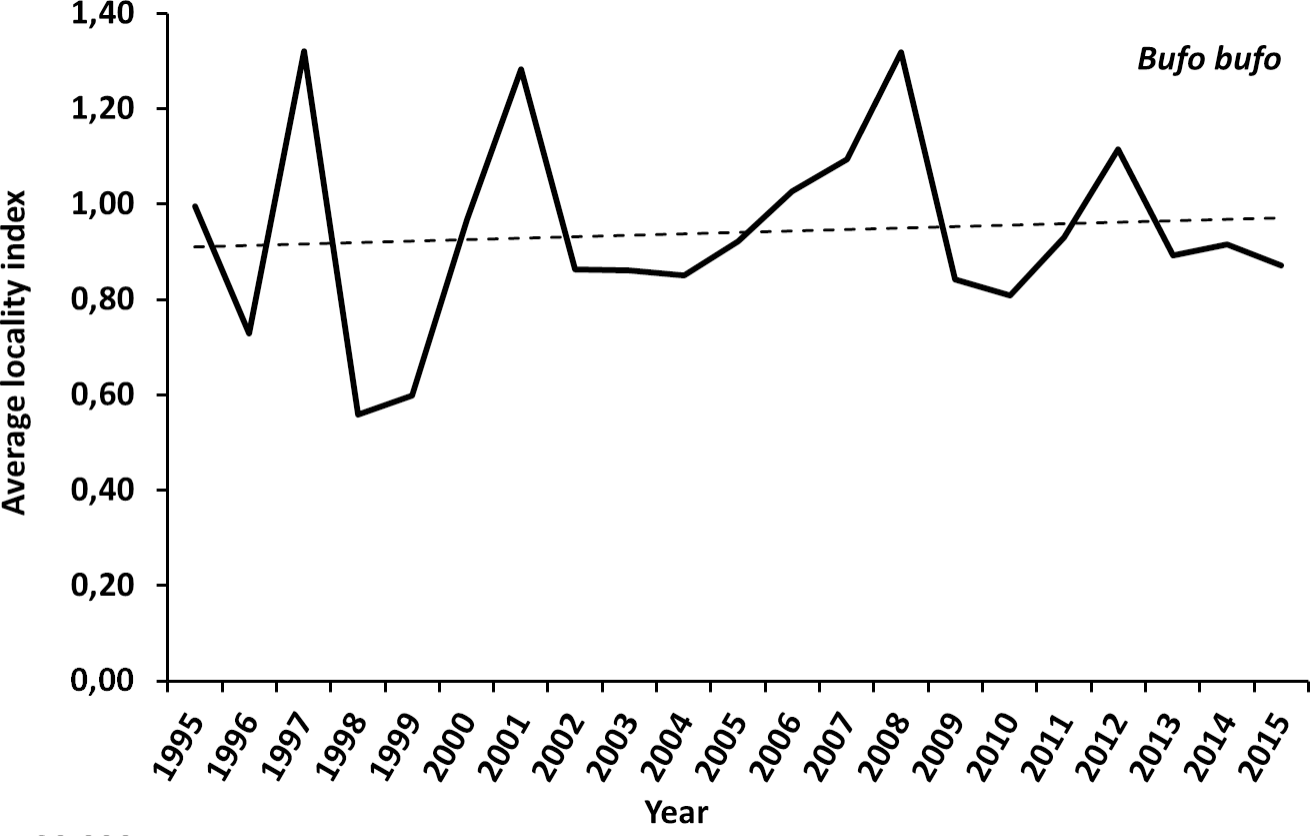
Average locality index (ALI) and linear regression of this index for the common toad. For better visualisation the ALI-base value of the first year (1995) was set to 1 (100%).

**Fig 4:**
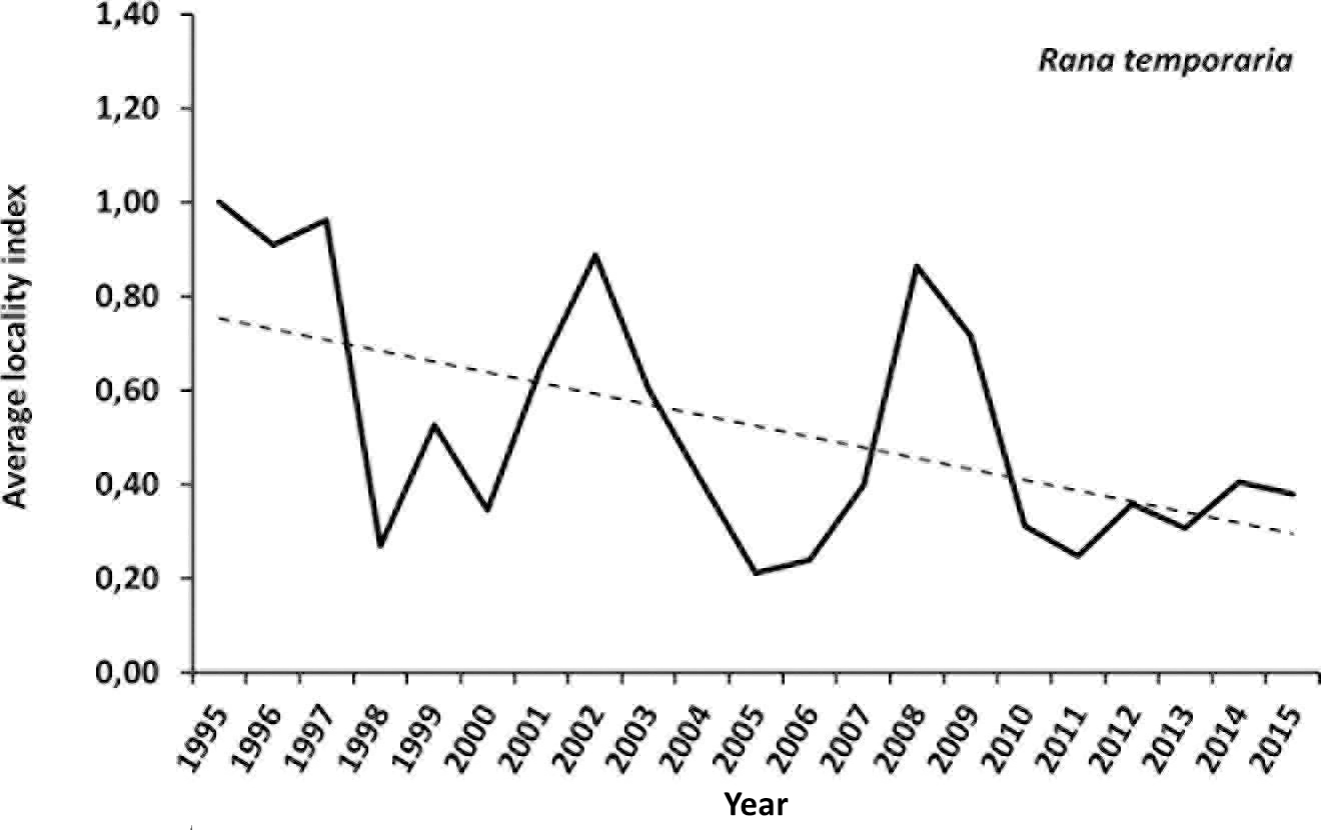
Average locality index (ALI) and linear regression of this index for the common frog. For better visualisation the ALI-base value of the first year (1995) was set to 1 (100%).

The average locality index for the common toad shows cyclic fluctuations. The overall linear regression for this species is slightly positive (+0.5% slope, see Fig 3). The average locality index for the common frog also shows cyclic fluctuations. Yet the overall linear regression is negative (-2% slope, see Fig 4).

### Analyses using TRIM

All test runs showed the same results for the overall slope of each species: The common toad being stable and common frog showing a moderate decline. Models using three geographical covariates have the lowest AIC, highest Wald test results and smallest standard deviations (see Tables 2 and 3). Due to the data being over-dispersed, the AIC-values are generally very high and Goodness-of-fit tests are invalid [24].

**Fig 5:**
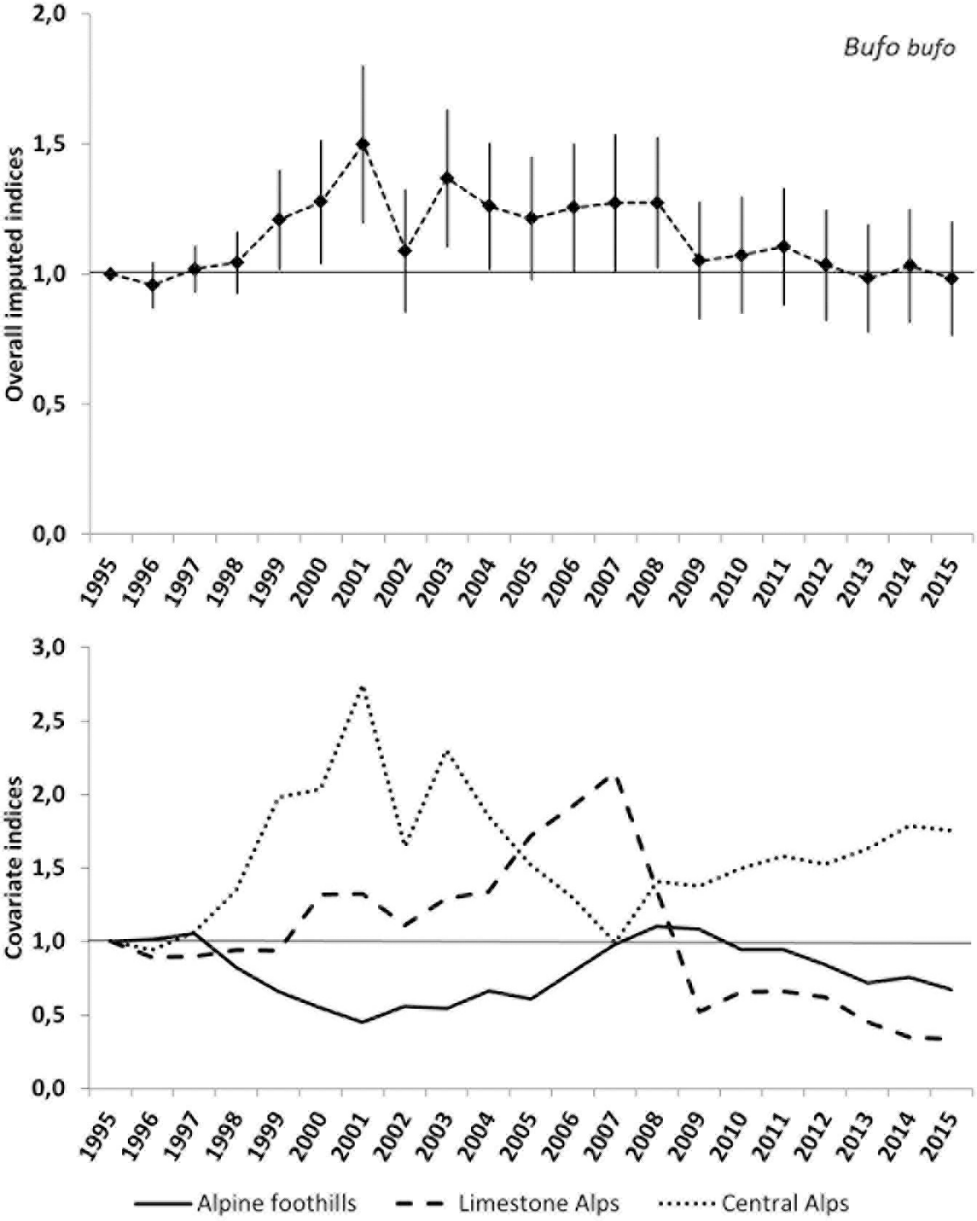
Plot of the overall imputed indices and standard errors as well as the plot of the imputed indices of the three geographical covariates for the common toad. The thin horizontal and undashed line indicates no change (index = 1).

The overall slope of the linear trend model for the common toad is stable. The indices show a positive trend between 1995 and 2001 and a sharp drop in the year 2002. Thereafter the indices are stable again until 2007 with a second drop in 2009. The early increase and the drop in 2002 were mainly driven by the population dynamics in the Central Alps. The second drop in 2009 was a consequence of a decline in the Limestone Alps. The imputed overall index of 2015 is 96 (±21) percent of the beginning value in 1995 (see Fig 5 and Table 4).

**Table 4:** Change rate of the 21-year-timeframe for the imputed indices based on the TRIM models for the overall trends as well as the three separate geographic regions.

	Bufo bufo		Rana temporaria	
	1995–2015	std. err.	1995–2015	std. err.
**Overall**	**-1.72%**	**±21.68**	**-82.67%**	**±4.2**
Alpine foothills	-32.55%	±28.21	-41.88%	±38.36
Limestone Alps	-66.39%	±10.58	-95.25%	±1.82
Central Alps	+75.50%	±34.39	-92.76%	±3.8

**Fig 6:**
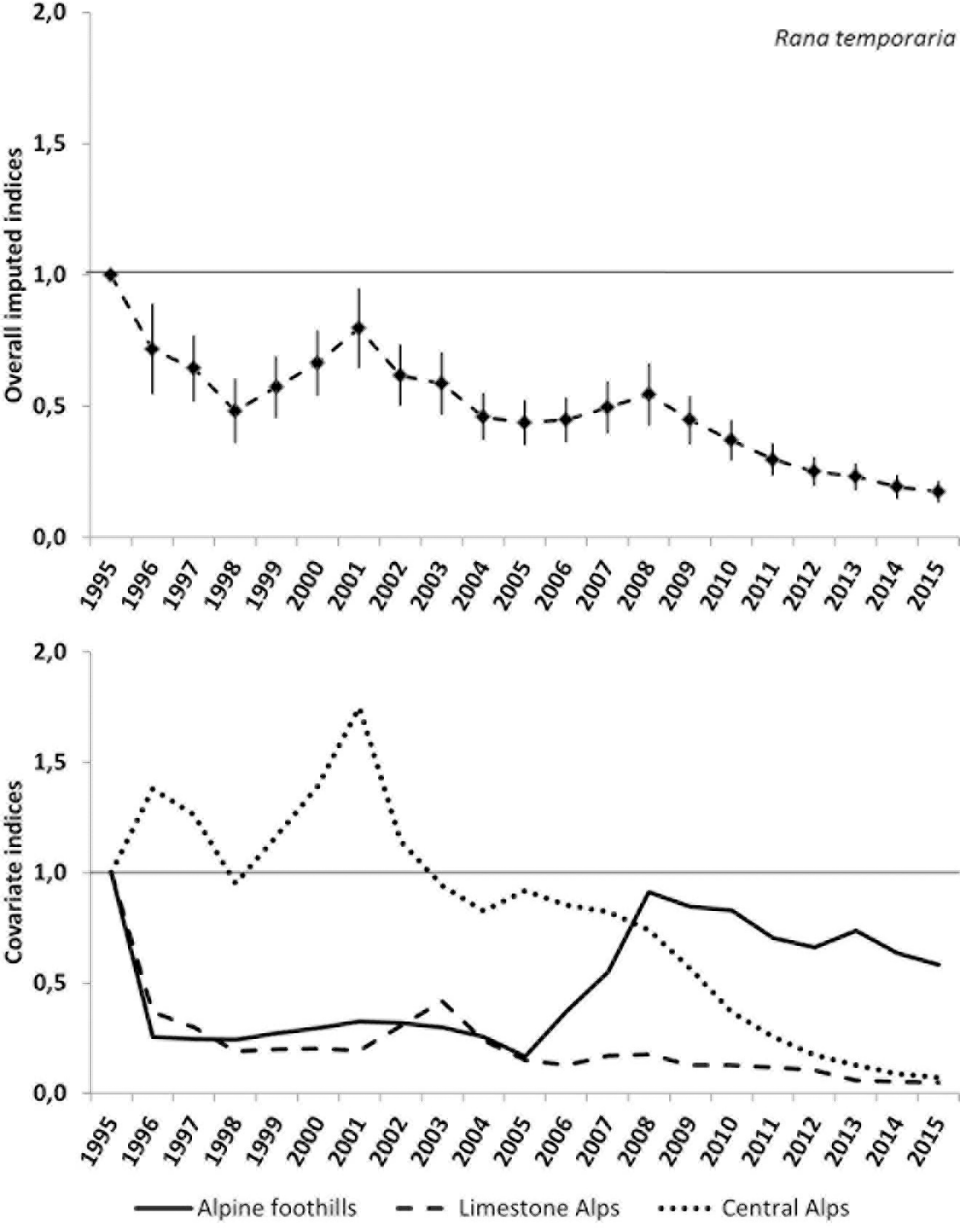
Plot of the overall imputed indices and standard errors as well as the plot of the imputed indices of the three geographical covariates for the common frog. The thin horizontal and undashed line indicates no change (index = 1).

The overall slope of the linear trend model for the common frog shows a decline (p<0.01). The indices show a negative trend between 1995 and 1998 followed by an increase until 2001. From then on the indices show a general negative trend only briefly interrupted by a moderate stabilization between 2004 and 2008. The first peak in 2001 is mainly driven by the population dynamics in the Central Alps. From 2005 to 2015 the three geographical regions show different population trends with an increase (until 2008) in the Alpine foothills and a strong decline in the Central Alps. The imputed overall index of 2015 dropped to 17 (±4) percent of the initial value in 1995 (See Fig 6 and Table 4).

TRIM calculations of a model with exclusion of the data from the eight migration sites, which were protected by tunnel systems, also showed a moderate decline (p<0.01) for the common frog. For the common toad the data set without those eight sites (which are those with the highest counts for common toads as well as for the common frog) is insufficient to calculate a TRIM model with the same parameters as in Table 2. The calculation of a simpler model with only time effects and no covariates also shows a stable population development for the common toad.

## 5. Discussion

The analyses of 21 years of amphibian fence counts presented in this study document a stable population development for the common toad and a decline of common frogs. Even though TRIM classifies this average decline of less than 5% per year as “moderate” rather than “steep” (see Table 3) the overall decline of 83% between 1995 and 2015 (21 years) must be summarized as substantial.

Population studies from Germany, Switzerland and Sweden show either highly variable or a stable trends for the common frog over the last decades [7, 10, 26, 27]. One survey from the British Isles detected an earlier decline of the common frog dating back to the 1960s [28]. The populations of common toads have been reported to be stable in different regions of Germany [29, 30], yet two studies estimated a dramatic decline for Italy, Switzerland and the United Kingdom also based on volunteer counts [9, 10]. Contrary to these findings, the common toad populations in Salzburg seem to be stable. The reduced population growth rates for the common frog after 2003 reported for Switzerland by Petrovan & Schmidt [10] correspond with our data (compare Fig 6).

The data analysed in this study were collected in populations which are fragmented by roads and whose spring migrations are protected by amphibian fences maintained by volunteers. Hence populations which are not fragmented by roads, as well as fragmented populations without maintenance are not recorded in this data set. Due to the dense road network of Salzburg, in fact 80% of all waterbodies which are inhabited by the common frog, are within a distance of 1.5 km to a public road [18]. This situation is most likely comparable to big parts of Central Europe. This means that populations in habitats not fragmented by roads would be exceptional for both species. Hence we have to assume that the reported trend is representative at least for the area studied.

### Statistical method

Concerning the overall trend at species level, both analyses (average locality index and TRIM) show the same result: a stable situation for the common toad and a decline for the common frog.

A possible flaw in our data regarding the TRIM-model is the imputation of missing values in those cases where the installation of a permanent tunnel-system ended the monitoring scheme at sites with previously declining numbers. Even though there is little evidence that tunnel systems cause positive long term effects on amphibian population trends [31, 32], their implementation may stabilize decreasing populations. The linear-regression model of TRIM however cannot take this effect into account and hypothetical counts are imputed for these sites based on their previous history as well as on the yearly trends at other sites [24]. Eight of the thirty-seven migration sites, which were included in this study, have been protected by a permanent tunnel-system in the last decade. Unfortunately no comparable monitoring data are available for these sites after installation.

A TRIM-based calculation of a model with a complete exclusion of the data from eight migration sites, which were protected by tunnel systems, showed the same results (moderate decline p<0.01) for the common frog. For the common toad, the data set without those eight sites (which are those with the highest counts for common toads as well as for the common frog) is insufficient to calculate a TRIM model with the same parameters as in Table 2. The calculation of a simpler model with only time effects and no covariates also suggested a stable population development for the common toad.

In this respect, the average-locality-index must be considered as the more conservative approach in terms of population decreases since it only incorporates the actually observed counts and no imputed values. The correlation between the ALI and the TRIM Indices show a significant correlation for the common frog (Pearson r = 0.687, p<0.01) but no correlation for the common toad (Pearson r = 0.331, p = 0.143) confirming the decline for the common frog and suggesting no time trend for the common toad. Therefore we consider that the general conclusion of differing trends for the two species is not a methodological artefact. The fact that data collected based on the same methodology, and by the same personnel, leads to different conclusions for the two species can be taken as evidence for a real biological effect and not a pure statistical artefact. Hence the dramatic decrease in common frogs presented here must be considered to be true.

### Road mortality

Road mortality is known to be one of the important factors driving population declines in amphibian species [32]. As explained above, all our data were collected in roadside counts using amphibian fences. These fences significantly reduce mortality of adults during the spawning migration. Yet they do not cover the return migration of adults or juveniles leaving the breeding ponds, which are of considerable importance for the long term population dynamics of amphibians [33, 34]. Therefore, the installation of tunnel systems, which also protect juvenile individuals, should be a better conservation tool than temporary fences. The fact, however, that the two species show different population trends, even though they have been studied at the same sites over the same period of time, means that traffic-induced mortality is probably not the main factor driving the observed decline of only one of the two studied species.

### Disease and climate change

In the context of amphibian decline, the roles of imported diseases, as well as climate change, are often presented in in contradictory ways. The reported decline of common frogs in the studied area (see Fig 6) overlaps with the first findings of Chytrid fungus in alpine areas of Austria [35]. In their study the authors tested 10 localities for *Batrachochytrium dendrobatidis* and found that the fungus was present at roughly 30% of the sites. Though they did not search at any sites in Salzburg, one of the locations in Tyrol is very close to the Salzburg border. At this site they tested one out of three common frogs positive for the fungus as well as three out of 20 water frogs (*Pelophylax* sp.). All over Austria roughly 23% of all tested common toads (53 individuals) and common frogs (3 individuals) were infected, yet none of the animals showed obvious signs of disease [35].

In their analyses of an amphibian mass mortality at a montane breeding site in Austria in 2004 and 2005 Sztatecsny and Hödl [36] did not find any evidence for chytridiomycosis. They rather emphasised that climatic factors might be the reason behind the sudden death of hundreds of common frogs and Alpine Newts (*Ichthyosaura alpestris*). The authors stressed that the reliance of amphibian populations on climatic, particularly temperature, conditions for phenological timing, as well as temporal breeding isolation between species, might have been the main factors behind the observed mass-mortalities. As models of climate change predict that the Alps are, and will be, more affected by global change than other areas [37], such temperature induced changes could be one of the reasons behind the observed decline, particularly of the alpine common frog populations, though it is hard to explain why this should affect one species more than the other.

### Differences between the two species

Even though the two species most often occur syntopically [18], they differ essentially in their ecology and habitat use. The common toad generally uses larger water bodies for reproduction and is even able to tolerate predatory fish [14, 26]. The common frog, on the other hand, has not only a reduced reproductive success in water bodies with fish, but also has more particular demands on the microclimate of its terrestrial habitat [15]. While the common toad is able to use a wide variety of terrestrial habitats and is often found in settlements or gardens, the common frog prefers wetter environments with high humidity, vegetation coverage and soil moisture, like wet meadows and forests with extensive agri- and silvicultural use [14, 15, 18]. Due to the humid climate on the northern slopes of the Alps, the natural landscape here was dominated by extensive bogs and wetlands [38]. Therefore human activities in Salzburg generally tend to drain big parts of the landscape and create drier habitats–a process that started centuries ago, but is still ongoing. Because of their specific biology, such developments might have a much greater effect on the common frog than on the common toad [14, 15]. Therefore we consider habitat destruction due to the intensification of land use, together with changes in silviculture and fisheries (mainly the lack of fish-free ponds), to be the main potential reasons behind the observed decline of the common frog.

### Geographical differences

Comparing geographical differences in population dynamics, the data for the common frog show a stronger decline in the two alpine regions than in the lowland area of Salzburg. This may be a consequence of the recent history of agricultural land use in Salzburg. The lowland areas of Salzburg have been used for more or less intensive farming for centuries [38]. It was not until the 1980s that first nature reserves in Salzburg were established, mainly located in the last remaining wetland habitats. On the other hand the intensification of agricultural practices in the lower areas–though still continuing–already reached high levels decades ago. We can therefore assume that the degradation or destruction of lowland habitats of the common frog dates back to times long before this study and any systematic amphibian census.

The intensive agricultural use of the alpine areas and mountain pastures started much later, usually initialized by infrastructural development (e.g. forest roads in alpine valleys). Ecological studies and aerial image analyses show that the intensification of land-use, particularly the drainage of wetlands in the alpine parts of Salzburg is an ongoing process (own observations see also [18, 39]). This observation is in agreement with findings by Stöcklin et al. [40] who concluded that in Switzerland currently alpine areas are undergoing extreme intensification processes comparable to changes in land-use outside the Alps during the 1960s and 1970s.

The different historic development of the landscape in the alpine region compared to the lowland areas fits with current observation of a decline of alpine common frog populations, comparable to declines outside the alpine areas decades ago. This corresponds with historical records of common frog spring migrations of up to 50.000 individuals until the 1950s in some alpine valleys, which can now no longer be observed [18].

The overall population indices for the common toad are stable, and there are no clear geographical trends visible (see Fig 5). However it seems that populations in the Central Alps are recently increasing, which is in marked contrast to the Limestone Alps. Yet the imputed indices for the three geographical covariate-groups show highly heterogeneous variations for the common toad, which also result in much higher standard deviations than for the common frog. This seems to reflect natural fluctuations of local sub-populations (maybe influenced by differing generation periods within different climatic regions [14]).

### Conclusions

Amphibian fences are not only an important conservation tool, they also can generate valuable data on population dynamics. The fact that we have to report a substantial decline of common frogs based on fence counts should not lead to dismissing such measures. However our results highlight the fact that these fences only protect small parts of the populations and that this is not enough to sustainably protect amphibians on a landscape level. We conclude that even when reducing road mortality with amphibian fences, other ecological factors lead to a continuous decrease of amphibian numbers. In our opinion the ongoing habitat loss seems to be the most important factor behind the described negative population trend of common frogs. But as Blaustein and Kiesecker [4] argue “amphibian population declines are caused by different abiotic and biotic factors acting together in a context-dependent fashion”. We can therefore not exclude other factors behind the observed trend and detailed ecological studies are needed to gain deeper insight into the reasons for this alarming decline of the common frog.

Even though the common frog obviously suffered severe losses in abundance throughout the last decades it is still the most widespread amphibian species in Salzburg and Central Europe. Yet the findings of this study make it necessary to re-evaluate the red-list status of the two species at regional level. Its wide distribution and ecological versatility, together with their higher palatability compared to other amphibians, makes the common frog an important part of the food chain. The different life cycle stages of this species are on the diet of almost every native predatory animal, from water insects up to birds and carnivorous mammals [15]. The recent decline of such a common species must therefore be considered a severe loss of ecosystem-function with potentially far reaching consequences [13]. Hence the loss of common frog biomass must be taken into account when discussing the decline of other predatory animals. We therefore emphasize that more conservation efforts, as well as research, must be focused on widespread and common species such as the common frog.

## Supporting information

S1 File Counts for Bufo bufo. Text file containing site numbers, years, counts and geographical covariates.
(TXT)

S2 File Counts for Rana temporaria. Text file containing site numbers, years, counts and geographical covariates.
(TXT)
